# Impact of El Niño-Southern Oscillation 2015-2016 on the soluble proteomic profile and cytolytic activity of Millepora alcicornis (“fire coral”) from the Mexican Caribbean

**DOI:** 10.7717/peerj.6593

**Published:** 2019-03-18

**Authors:** Norma Olguín-López, Víctor Hugo Hérnandez-Elizárraga, Rosalina Hernández-Matehuala, Andrés Cruz-Hernández, Ramón Guevara-González, Juan Caballero-Pérez, César Ibarra-Alvarado, Alejandra Rojas-Molina

**Affiliations:** 1 Posgrado en Ciencias Químico Biológicas–Facultad de Química, Universidad Autónoma de Querétaro, Querétaro, Querétaro, Mexico; 2 Laboratorio de Investigación Química y Farmacológica de Productos Naturales, Facultad de Química, Universidad Autónoma de Querétaro, Querétaro, Querétaro, Mexico; 3 Laboratorio de Biología Molecular–Escuela de Agronomía, Universidad De la Salle Bajío, León, Guanajuato, México; 4 C.A Ingeniería de Biosistemas–Facultad de Ingeniería-Campus Amazcala, Universidad Autónoma de Querétaro, Querétaro, Querétaro, Mexico; 5 Facultad de Química, Universidad Autónoma de Querétaro, Querétaro, Querétaro, Mexico

**Keywords:** *Millepora alcicornis*, Reef forming cnidarians, Hydrozoan cnidarians, Proteomic profile, Soluble proteome, Cytolysins, Fire coral, Global warming, Bleaching

## Abstract

Reef-forming cnidarians are extremely susceptible to the “bleaching” phenomenon caused by global warming. The effect of elevated seawater temperature has been extensively studied on Anthozoans; however, to date the impact of thermal stress on the expression of genes and proteins in Hydrozoan species has not been investigated. The present study aimed to determine the differential proteomic profile of *Millepora alcicornis*, which inhabits the Mexican Caribbean, in response to the El Niño-Southern Oscillation 2015–2016. Additionally, the cytolytic activity of the soluble proteomes obtained from normal and bleached *M. alcicornis* was assessed. Bleached specimens showed decreased symbiont’s density and chlorophyll a and c2 levels. After bleaching, we observed a differential expression of 17 key proteins, tentatively identified as related to exocytosis, calcium homeostasis, cytoskeletal organization, and potential toxins, including a metalloprotease, a phospholipase A2 (PLA2), and an actitoxin. Although, some of the differentially expressed proteins included potential toxins, the hemolytic, PLA2, and proteolytic activities elicited by the soluble proteomes from bleached and normal specimens were not significantly different. The present study provides heretofore-unknown evidence that thermal stress produces a differential expression of proteins involved in essential cellular processes of Hydrozoan species. Even though our results showed an over-expression of some potential toxin-related proteins, the cytolytic effect (as assessed by hemolytic, PLA2, and caseinolytic activities) was not increased in bleached *M. alcicornis*, which suggests that the cytolysis is mainly produced by toxins whose expression was not affected by temperature stress. These findings allow hypothesizing that this hydrocoral is able to prey heterotrophically when suffering from moderate bleaching, giving it a better chance to withstand the effects of high temperature.

## Introduction

Coral reefs play a critical role in marine ecology and human sustainability (Jain, Sonawane & Mandrekar, 2008; Mumby & Van Woesik, 2014; Anthony, 2016; Williams et al., 2016). Moreover, the organisms that constitute these ecosystems are considered a rich source of novel bioactive agents with considerable pharmaceutical and biotechnological potential, since they produce a great variety of molecules with unique structural features (Jain, Sonawane & Mandrekar, 2008; Cragg & Newman, 2013; Mayer et al., 2016; Li, Ding & Li, 2017). Organisms of the genus *Millepora*, which belong to the class *Hydrozoa*, are considered the second most important reef forming species, after scleractinian hard corals of the class Anthozoa (Lewis, 2006; Ruiz-Ramos, Weil & Schizas, 2014). In addition to their ecological importance, these organisms, commonly known as “fire corals,” induce local and systemic toxic effects in humans (Lewis, 2006; Rojas-Molina et al., 2012).

Unfortunately, coral reefs are extremely vulnerable to the stress related to greenhouse gas emissions, mainly ocean warming (Lesser, 2007; Baird et al., 2009; Mumby & Van Woesik, 2014; Lough, 2016). Elevated seawater temperature provokes disturbances that can seriously affect and break down the homeostatic capacity of coral reefs to overcome stressors (Mora, Graham & Nyström, 2016). One of the most devastating consequences of global warming is coral bleaching, in which corals and hydrocorals lose their photosynthetic symbiotic algae of the genus *Symbiodinium* or their pigments, which exposes the white exoskeleton composed of calcium carbonate (Hoegh-Guldberg, 1999; Lesser, 2006; Bonesso, Leggat & Ainsworth, 2017; Neal et al., 2017).

The frequency and seriousness with which this phenomenon occurs have increased in recent years. Massive bleaching events have been recorded in all the tropical regions of the world in 1987, 1998, 2003, 2005, and 2010 (Eakin et al., 2010; Hoegh-Guldberg & Bruno, 2010; Heron et al., 2016). In fact, during 2014–2017, the worst documented bleaching event was observed (Eakin et al., 2017; Hughes et al., 2017b, 2018). Numerous investigations have demonstrated that bleaching is deleterious to coral reefs. Bleaching events have caused massive damage to coral reefs around the world, with very severe effects on the balance of biodiversity in marine tropics. This phenomenon causes a general deterioration in reef health, as it provokes an increase in coral diseases, a decay in reef calcification, breakdown of reef framework by bioeroders, and loss of essential habitat for related reef organisms (Baker, Glynn & Riegl, 2008; Lesser, 2011; Grottoli et al., 2014; Okazaki et al., 2016; Hughes et al., 2017a). Some climate models predict that if CO_2_ emissions continue at the current rate, bleaching events will increase its frequency and severity, seriously threatening the survival of coral reefs (Houlbreque & Ferrier-Pagès, 2009; Lesser, 2011; Mumby & Van Woesik, 2014; Lough, 2016; Neal et al., 2017; Hughes et al., 2018).

Studies that have addressed the etiology and effects of bleaching have focused on Anthozoan species. To date there are no reports regarding the impact of thermal stress on the expression of genes and proteins of Hydrozoan species. Genomic, transcriptomic, and proteomic approaches have revealed that heat stress causes a differential pattern in the expression of genes and proteins from various cellular processes, including oxidative stress response, Ca^2+^ homeostasis, cytoskeletal organization, protein synthesis, apoptosis, endo-exophagocytosis, and immune response. Also, the differential expression of heat shock proteins and transcription factors has been observed (Bay & Palumbi, 2015; Pinzón et al., 2015; Raina et al., 2015; Seneca & Palumbi, 2015; Weston et al., 2015; Maor-Landaw & Levy, 2016; Oakley et al., 2016, 2017; Ricaurte et al., 2016; Sloan & Sawyer, 2016; Huang et al., 2017; Ruiz-Jones & Palumbi, 2017; Traylor-Knowles et al., 2017; Mayfield et al., 2018a).

It is generally accepted that cnidarian-*Symbiodinium* symbiosis is fundamental for the formation of coral structures, since algae carry out photosynthesis and transfer more than 50% of their photosynthetic products to the cnidarian host (Houlbreque & Ferrier-Pagès, 2009; Davy, Allemand & Weis, 2012; Fransolet, Roberty & Plumier, 2012; Douglas, 2003; Venn, Loram & Douglas, 2008; Yellowlees, Rees & Leggat, 2008). Up to date, the role of heterotrophic carbon (C) input in the resilience and recovery of bleached reef forming cnidarians is still controversial. It has been observed that during bleaching certain species can increase their heterotrophic feeding to maintain and restore energy reserves (Grottoli, Rodrigues & Palardy, 2006; Aichelman et al., 2016). However, some studies suggest that heterotrophically derived fixed carbon does not completely attenuate C budget imbalance (Levas et al., 2016; Tremblay et al., 2016).

Undoubtedly, after a bleaching event the central metabolism of both symbiotic partners is significantly altered (Ricaurte et al., 2016; Hillyer et al., 2017, 2018; Oakley et al., 2017; Mayfield et al., 2018a), and the contribution of heterotrophy in the response of cnidarian-*Symbiodinium* symbiosis to elevated temperature is still unclear (Grottoli, Rodrigues & Palardy, 2006; Levas et al., 2016; Aichelman et al., 2016; Tremblay et al., 2016). Considering that toxins are essential in prey capture and digestion, it is very likely that thermal stress, which provokes bleaching, alters their expression in cnidarians. Therefore, assessment of the effect of thermal stress on the expression of cnidarian toxins used to capture preys is fundamental to understand if heterotrophy constitutes a significant survival mechanism of reef-forming cnidarians after a bleaching phenomenon. In this context, the aim of the present study was to determine changes in the soluble proteomic profile and cytolytic activity of *Millepora alcicornis* (“fire coral”) that suffered bleaching during El Niño-Southern Oscillation (ENSO) 2015–2016 in the Mexican Caribbean Sea.

## Materials and Methods

### Specimen collection

Hydrocoral fragments were collected by removing small portions from the edges of three normal and three visibly bleached *M. alcicornis* colonies in November 2016, within the third global-scale mass bleaching event ever documented (Hughes et al., 2017b, 2018). Hydrocorals were obtained at depths of 4–10 m. The possibility of sampling genetic clones was reduced by collecting fragments that were at least 10 m apart in the area known as “La Bocana Chica” that belongs to the Parque Nacional Arrecife de Puerto Morelos (Quintana Roo, México). Collection permit was issued by Secretaría de Agricultura, Desarrollo Rural, Pesca y Alimentación (permit number PPF/DGOPA-139/15). Specimens were frozen in liquid nitrogen and transported to our laboratory at the Universidad Autónoma de Querétaro.

### Determination of cell density in host tissues

Bleached and normal fragments of *M. alcicornis* were cut into one cm^2^ squares, subsequently, tissues were fixed with 10% buffered formalin during 3 days. Thereafter, samples were decalcified in a 5% solution of HCl for 5 days. The decalcifying solution was replaced daily. After this treatment, tissues were homogenized in a Glass-Col tissue homogenizer system during 2 min at 70 rpm, and centrifuged at 2,400 rpm. The resulting pellet was resuspended in 70% ethanol. A total of 10 μl-aliquots of this homogenate were used to count *Symbiodinium* cells using a Neubauer hemocytometer. Cell counts were performed in triplicate and were standardized in relation to the total surface area of one cm^2^.

### Determination of chlorophyll content in *Symbiodinium*

Chlorophyll a and c2 contents were quantified employing the method of Shirur et al. (2014). Briefly, normal and bleached hydrocoral fragments were incubated in the dark for 24 h at 4 °C with a mixture of acetone: dimethyl sulfoxide (95:5 v/v). Chlorophylls a (Chl a) and c (Chl c2) contents were calculated using the equations of Jeffrey & Humphrey (1975) and were then standardized to *M. alcicornis* surface area.

### Soluble proteome extraction

Nematocyst discharge and soluble proteome extraction from normal and bleached *M. alcicornis* fragments were accomplished by stirring the hydrocoral fragments in deionized water (pH 7) at 4 °C for 24 h. The aqueous extracts obtained, containing the soluble proteomes, were centrifuged at 12,000 rpm for 15 min at 4 °C. Subsequently, the supernatants were filtered through a 0.45 μm pore filter (Millipore Ltd, Hertfordshire, England). The filtrates were lyophilized and stored at −70 °C. For proteomic analysis, the lyophilized filtrates were dissolved in 100 μl of deionized water employing microtubes for centrifuge. Thereafter, proteins were cleaned using a Ready Prep 2-D Cleanup Kit (Bio-Rad, Hercules, CA, USA). The protein concentration in both soluble proteomes was determined using a 2D-Quant Kit (GE Healthcare Life Sciences, Uppsala, Sweden).

### SDS–PAGE

Sodium dodecyl sulfate–polyacrylamide gel electrophoresis (SDS–PAGE) was carried out as previously described (Schägger & Von Jagow, 1987). Samples of normal and bleached *M. alcicornis*-soluble proteomes containing 80 μg of protein were run in 18% polyacrylamide gels at 150 V for 2 h at 4 °C, using tris-glycine as buffer. Protein bands were visualized with BioSafe Coomassie blue G-250 dye (Bio-Rad, Hercules, CA, USA). Molecular weights were determined using Precision Plus Protein™ standards (Bio-Rad, Hercules, CA, USA).

### First-dimension step, isoelectric focusing

One milligram of protein from the soluble proteomes of normal and bleached hydrocorals was mixed with rehydration buffer (8M Urea, 2% CHAPS, 50 mM dithiothreitol (DTT), 0.2% BioLyte® 3/10 Ampholyte) overnight on immobilized pH gradient using 11 cm ReadyStrip™ IPG strips pH 3–10 (Bio-Rad, Hercules, CA, USA), which were then subjected to first-dimension isoelectric focusing (IEF) in a PROTEAN® i12™ IEF System (Bio-Rad, Hercules, CA, USA) for a total of 20,000 Vh, as previously described (Ricaurte et al., 2016).

### High-resolution two-dimensional gel electrophoresis

After completion of the IEF, and prior to running the second dimension, strips were firstly submerged in equilibration buffer I (urea 6M, 2% SDS, Tris–HCl 0.05M, pH 8.8, 50% glycerol, and 2% (w/v) DTT) to reduce the S–S bonds, and subsequently alkylated in equilibration buffer II (urea 6M, 2% SDS, Tris–HCl 0.05M, pH 8.8, 50% glycerol and 2.5% (w/v) Iodoacetamide). The second-dimensional separation was carried out in 18% SDS-polyacrylamide gels at 150 V for 2 h at 4 °C, using tris-glycine buffer. Gels containing the soluble proteomes of normal and bleached *M. alcicornis* were stained with BioSafe™ Coomassie blue G-250 dye (Bio-Rad, Hercules, CA, USA).

### Image analysis

Images of the stained gels were captured in a ChemiDoc™ MP (Bio-Rad, Hercules, CA, USA). imaging system at 600 dpi resolution. Spot detection, matching, and fold changes were determined with the PD-Quest™ software (Bio-Rad, Hercules, CA, USA), version 8.0.1. Protein spots with fold change ≥2 between proteomes of normal and bleached specimens were regarded as differentially expressed. These spots were selected and marked for excision. All experiments were performed in triplicates.

### Protein in-gel digestion and data analysis

Selected spots were manually excised and faded with ACN: 50 mM NH_4_HCO_3_ (50:50 v/v). Protein spots were processed for protein identification at Instituto Nacional de Medicina Genómica, México. Protein samples were subjected to enzymatic digestion for 18 h at 37 °C with mass spectrometry-grade-trypsin (V528A; Promega, Madison, WI, USA). Peptides obtained from the digestion (ACN:H_2_O:formic acid 50:45:5 v/v) were extracted and the volume of samples was reduced in a concentrator Eppendorf 5301 (Eppendorf, Hamburg, Germany). Samples were desalted by a C18 column (ZipTipC18 15 μm; Millipore, Bedford, MA, USA). Subsequently, a MALDI-TOF/TOF tandem mass spectrometry analysis was carried out to identify the selected proteins. Afterward, each protein sample was co-crystallized in plates using an α-Cyano-4-hydroxycinnamic acid matrix. MALDI-TOF/TOF experiments were carried out on a 4800 MALDI-TOF/TOF Analyzer (Applied Biosystems, Foster City, CA, USA) equipped with a 200 Hz, 355 nm Nd:YAG laser, linked to 4,000 series Explorer software version 3.5.3. The mass spectrometer was operated in positive ion mode and externally calibrated using a peptide mass standard. The laser source was set to 2,500–2,800 for MS and 3,500–3,800 for MS/MS acquisition. MS spectra were recorded in positive ion reflector mode with 25 laser shots. Precursor ions were automatically selected for fragmentation, which was carried out employing a collision energy of two kV, using air as the collision gas (pressure, 2 × 10^–6^ torr), with the accumulation of 400 shots. Mass spectra were acquired within a mass range of 800–4,000 Da (m/Z). The parental ion of Glu1-Fibrino-Peptide B, diluted in the matrix (1.3 pmol/μl/spot), was employed for internal calibration at *m*/*z* = 1,570.690 Da. The most intense ion signals per spot position having an S/N >20 were chosen for MS/MS acquisition. Following MALDI-TOF/TOF analysis, a search was performed, employing the Protein Pilot™ software version 2.0 (Applied Biosystems, Foster City, CA, USA). Carbamidomethylation of cysteine was set as fixed modification. The Basic Local Alignment Search Tool (BLASTp) (https://blast.ncbi.nlm.nih.gov/Blast.cgi) was used for identification of homolog sequences. Database was set to non-redundant protein sequences, using Cnidaria as “organism parameter search.” Similar sequences were aligned with the multiple sequence alignment program ClustalW2 (https://www.ebi.ac.uk/Tools/msa/clustalw2/). The criterion for the selection of protein spots considered the comparison of experimental isoelectric point (pI), molecular weight, and sequence coverage of proteins with that of the theoretical pI and molecular weight of the proteins detected on the 2DE-PAGE gels.

### Evaluation of cytolytic activity of the soluble proteomes from normal and bleached *M. alcicornis* specimens

#### Comparative hemolytic activity of the soluble proteomes from normal and bleached M. alcicornis specimens

Hemolytic activity of the soluble proteomes from normal and bleached *M. alcicornis* specimens was monitored according to a method previously described with some modifications (Hernández-Matehuala et al., 2015). Briefly, samples for the assay contained a mixture (one mL) of Alsever’s solution (120 mM D-glucose, 30 mM sodium citrate, seven mM NaCl, and two mM citric acid; pH 7.4) with 50 μl of a 1% suspension of erythrocytes from rat and the required volume of each soluble proteome. These samples were incubated at 37 °C for 30 min. After centrifugation at 1,430 g for 4 min at 4 °C, the absorbance at 415 nm of the supernatant fluid containing the hemoglobin released from lysed erythrocytes was measured in a Benchmark Plus microplate spectrophotometer (Bio-Rad Laboratories, Hercules, CA, USA). Each experiment was normalized with respect to complete hemolysis, which was measured by diluting the erythrocyte sample in deionized water instead of Alsever’s buffer. One hemolytic unit (HU50) was defined as the amount of protein sample required to cause 50% hemolysis. The hemolytic activity was plotted in GraphPad Prism 6.0 (GraphPad Software, San Diego, CA, USA).

#### Comparative phospholipase A2 activity of the soluble proteomes from normal and bleached *M. alcicornis* specimens

The phospholipase A2 (PLA2) activity of the soluble proteomes from normal and bleached *M. alcicornis* specimens was determined using a secretory PLA2 colorimetric assay kit (Cayman Chemical, Ann Arbor, MI, USA). The assay uses the 1,2-dithio analog of di-heptanoyl phosphatidylcholine as substrate. Free thiols generated upon hydrolysis of the thioester bond at the sn-2 position by PLA2 were detected using DTNB (5,5′-dithio-bis-(2- nitrobenzoic acid)). Color changes were monitored by a Benchmark Plus microplate spectrophotometer (Bio-Rad Laboratories, Hercules, CA, USA) at 414 nm, sampling every minute for 10 min. The PLA2 activity was expressed as micromoles of hydrolyzed substrate per minute per milligram of protein. The PLA2 activity was calculated using GraphPad Prism 6.0 (GraphPad Software, San Diego, CA, USA).

#### Comparative proteolytic activity of the soluble proteomes from normal and bleached M. alcicornis specimens

The proteolytic activity of the soluble proteomes from normal and bleached *M. alcicornis* specimens was determined according to a method previously described with some modifications (Nagaraju et al., 2006). Briefly, soluble proteomes (50 μg protein) were incubated separately with one mL of substrate (2% casein in 50 mM Tris, 100 mM NaCl, five mM CaCl_2_, pH 8.8) for 2 h and 30 min at 37 °C. Undigested casein was precipitated by adding 1.5 mL of 0.44M trichloroacetic acid. The digested casein in the supernatant (one mL) was determined using the Folin–Ciocalteu’s reagent. One unit of activity was defined as the amount of enzyme required to cause an increase in the optical density by 0.01 at 660 nm/min. Data obtained from the proteolytic assay were plotted using GraphPad Prism 6.0 (GraphPad Software, San Diego, CA, USA).

### Data analysis and statistics

Results are expressed as mean ± S.E.M. from *n* = 3 experiments. In the case of the hemolytic activity, concentration-response curves (CRC) were repeated three times employing different rats. CRC were plotted and fitted to the Boltzmann equation, using the data analysis and graphics software GraphPad Prism 6.0 (GraphPad Software, San Diego, CA, USA). Means ± S.E.M. obtained from cell density, chlorophyll content, hemolytic, PLA2, and caseinolytic assays were compared using an unpaired Student’s *t*-test. In all cases, statistical significance is indicated by *p* < 0.05.

## Results

**Figure 1:**
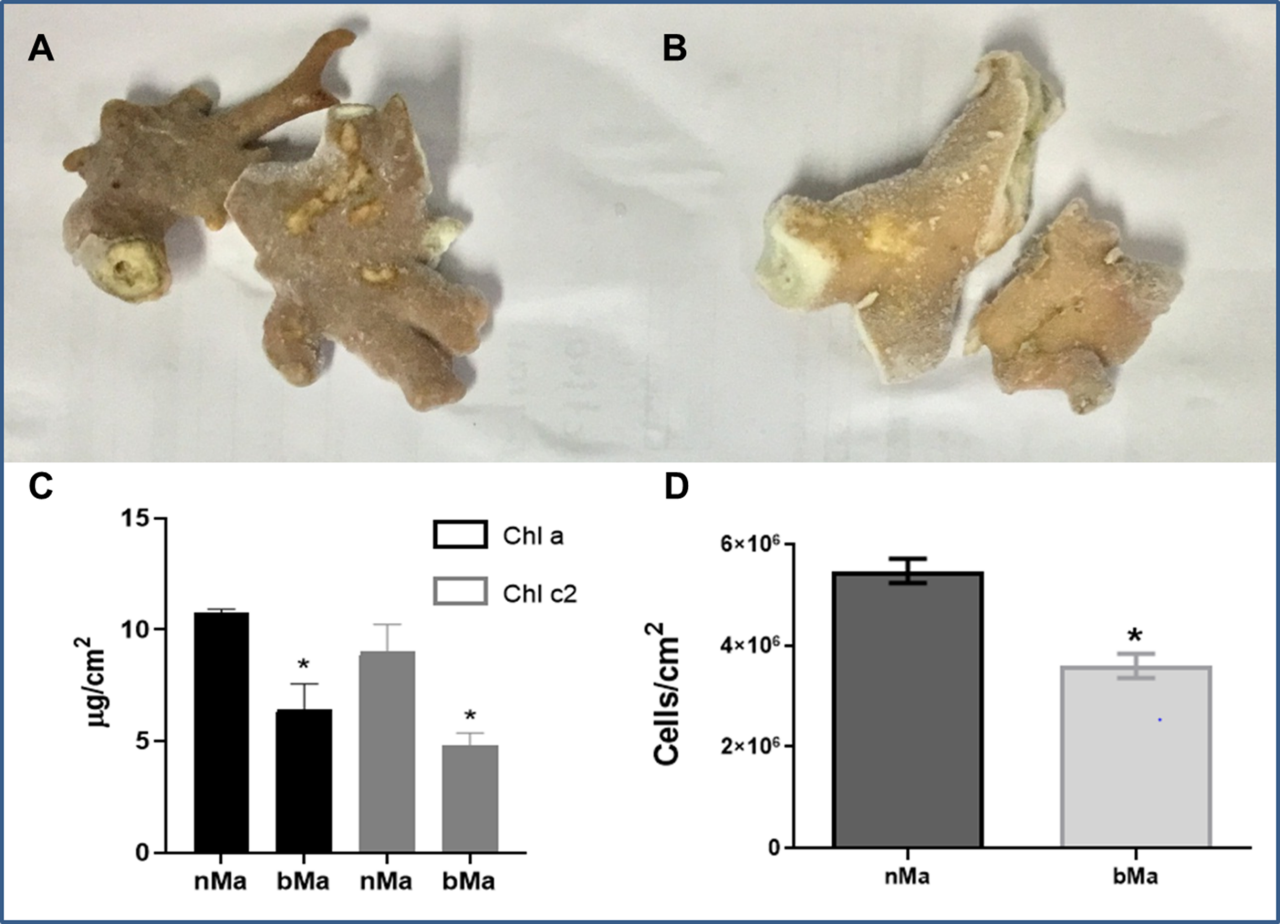
Normal (A) and bleached (B) fragments from *M. alcicornis*. (C) Chlorophyll a (Chl a) and chlorophyll c (Chl c2) content per cm^2^. (D) Symbiont´s density quantified for normal and bleached specimens. Data are mean ± SEM. *Indicate significant difference. Photographs from normal and bleached specimens were taken by Norma Olguín-Lopez and Víctor Hugo Hernández-Elizárraga.

Normal (Fig. 1A) and visibly discolored (Fig. 1B) fragments of the “fire coral” *M. alcicornis* were collected in the Mexican Caribbean in November 2016. Bleaching caused significant decreases in chlorophyll a and chlorophyll c2 content (Fig. 1C), and cell density of symbionts (Fig. 1D).

**Figure 2:**
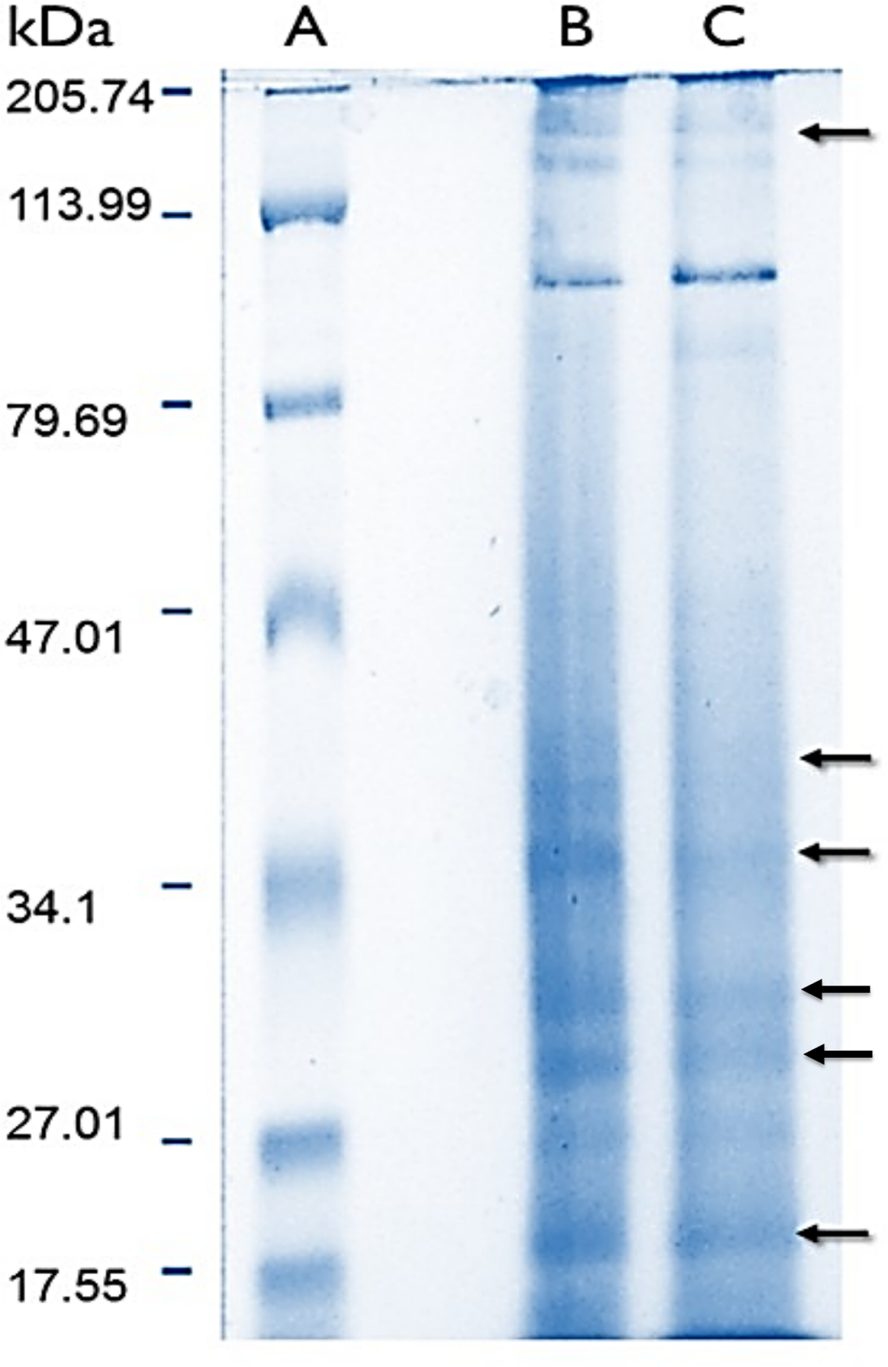
Electrophoresis profiles of the soluble proteomes from normal (A) protein molecular weight marker (B) and bleached (C) specimens of *M.* alcicornis. Samples (80 μg of protein) were separated by one dimensional SDS–PAGE using 18% (w/v) polyacrylamide under non-reducing conditions. Protein bands were stained using Coomassie Blue R-250. Arrows indicate protein bands in which differences were observed.

Protein content in lyophilized soluble proteomes from normal and bleached specimens were 21.12 and 13.96 μg of protein per mg of lyophilizate, respectively. The SDS–PAGE analysis revealed that proteomes from normal and bleached hydrocorals have different protein profiles and contained proteins with a broad range of molecular weights, ranging in size from 10 to 200 kDa (Fig. 2).

**Figure 3:**
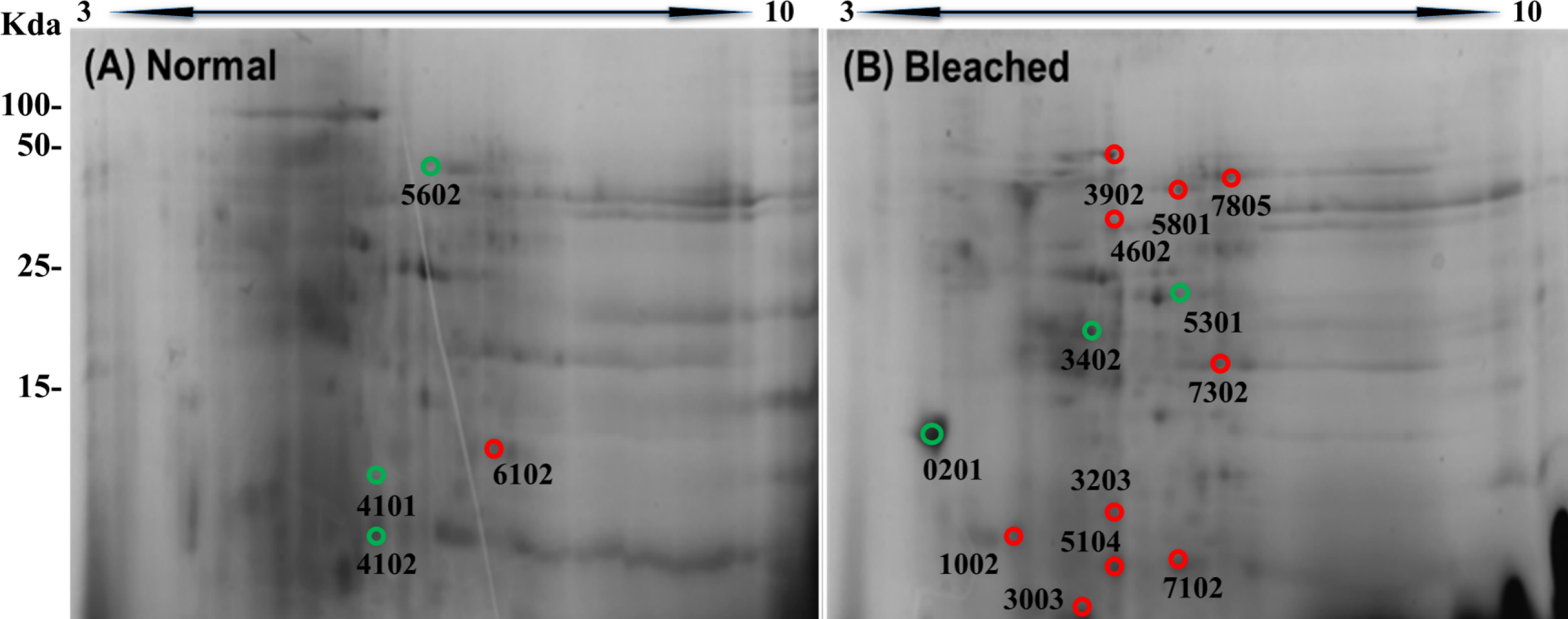
Two-dimensional gels of the soluble proteomes from normal (A) and bleached (B) *M. alcicornis* specimens. Up-regulated proteins are marked with green circles and down-regulated proteins are marked with red circles. Protein spots with differential expression were selected based on a fold change ≥2.

2DE-PAGE analysis allowed the detection of 52 and 75 protein spots in the proteomes of normal (Fig. 3A) and bleached hydrocorals (Fig. 3B), respectively. 2DE-PAGE gels showed well-resolved protein spots with molecular masses between 10 and 50 kDa and pI values ranging from 4 to 8. A total of 17 proteins were differentially expressed in bleached hydrocorals with respect to normal specimens, six of these proteins were up-regulated after bleaching (Fig. 3A), while eleven were down-regulated (Fig. 3B). Analysis by MALDI-TOF/TOF, the ProteinPilot software, and BLASTp was carried out with eight of the differentially expressed proteins (Table 1). The other protein spots were not examined due to their low concentration.

**Table 1:** *M. alcicornis* proteins identified by MALDI-TOF/TOF, the ProteinPilot software, and BLASTp analysis.

Spot	Protein name	Accession no.	Peptides	No of peptides matched	*m*/*z*	Mw/pI	Sequence coverage
201	Calmodulin	XP_020603574.1	EAFSLFDK DGNGFISAAELR VFDKDGNGFISAAELR EAFSLFDKDGDGTITTK ADQLTEEQIAEFK EADIDGDGQVNYEEFVK	6	956.5549 1,249.6982 1,738.9722 1,845.0011 1,563.8358 1,927.9585	16.89/3	24.5%
1002	Disintegrin and metalloproteinase domain-containing protein 7	KII70418.1	TLGENGKEFSK EFLLIFR	2	1,223.7189 937.6454	10.47/4.02	7.5%
3402	DELTA-actitoxin-Ate1a-like	XP_020897118.1	FLQTEMDK ELGVDYFLERR EAHLILFLVNAR RIKLKYAHLGSSNPP	4	1,028.5859 1,378.8389 1,378.8082 1,713.0261	18.55/4.39	22.7%
4102	Phospholipase A2	XP_004208832.1	TLGLPTAPYVEALSFAR QDDERANYAK	2	1,806.0854 1,211.6534	11.61/4.99	19.8%
7102	ATP-dependent Zinc metalloprotease	KII60716.1	NRARALINIAHPK QFISSLEDAETIKSFNNLR KNAGKAINAHLIPK QKAENILSFLQK ALLLLKDFDSVPPVVR LSVNTPNNRQPLTITVSTKK	6	1,474.918 2,212.2581 1,474.922 1,418.8232 1,799.0536 2,211.2761	12.56/5.98	68.9%
7805	Component 3 exocytosis complex	KII70395.1	NFAKLSQSLETLDSQLNLR TSGDKGKTSPR	2	2,211.3179 1,182.6781	70.09/6.15	3.9%
5301	Actin	XP_020600429.1	SYELPDGKVITIGNER	1	1,792.0223	32.02/5.41	5.6%
3203	Collagen alpha	XP_020603217.1	GSAGPPGATGFPGAAGR	1	1,459.8524	14.41/4.83	4.3%

**Note:**

The protein spots marked in red showed down-regulation in bleached *Millepora alcicornis*, while the protein spots marked in green showed up-regulation expression.

Our results showed that calmodulin (CaM), actin, and two potential toxins (DELTA-actitoxin-Ate1a-like and a PLA2) were up-regulated after bleaching, whereas component 3 exocytosis complex, collagen alpha, a disintegrin metalloproteinase domain-containing protein 7, and an ATP-dependent zinc metalloprotease were down-regulated. All proteins were identified on the basis of manual annotations using BLASTP to identify proteins with homology to Cnidarian proteins described in the NCBI database.

On the other hand, the soluble proteomes from normal and bleached specimens elicited a concentration-dependent hemolysis. In addition, both soluble proteomes displayed PLA2 and caseinolytic activities. No statistically significant difference was observed between the hemolytic and enzymatic activities produced by the proteome from normal specimens and those induced by the proteome from bleached hydrocorals (Table 2).

**Table 2:** Protein content, hemolytic, PLA2, and caseinolytic activities of the soluble proteomes from normal and bleached *M. alcicornis* specimens.

Extracts	Protein content (μg protein/mg lyophilized)	Hemolytic activity (Hu_50_, μg protein/ml)	PLA2 activity (μmol/min/mg)	Caseinolytic activity (U/min/μg protein)
**Normal**	21.12 ± 1.1	0.1343 ± 0.02	41.37 ± 2.1	27.57 ± 1.43
**Bleached**	13.96 ± 1.4*	0.09061 ± 0.005	49.11 ± 0.9	30.82 ± 2.05

**Notes:**

Each value is expressed as the mean ± S.E.M. (*n* = 3).

* *p* < 0.05 compared to control.

PLA2, phospholipase A2.

## Discussion

In recent decades, increases in the frequency and intensity of coral bleaching events have resulted in declines in coral cover worldwide (Roff, Zhao & Mumby, 2015; Lough, 2016). Accordingly, these damaged ecosystems have suffered a dramatic “phase shift,” in which habitats dominated by reef-forming organisms are now dominated by macroalgae and low-relief corals (Jackson et al., 2014; Cramer et al., 2017). In fact, coral reefs are considered the most degraded ecosystems on the planet due to climate change (Eakin et al., 2010; Roff, Zhao & Mumby, 2015; Cramer et al., 2017). During 2014–2017, record high temperatures triggered the third global scale event of coral bleaching ever registered (Eakin et al., 2017; Hughes et al., 2017b, 2018). It is estimated that 30% of the reef areas that were monitored after the ENSO 2015–2016 showed severe bleaching (Hughes et al., 2018), and the Caribbean coral reefs were seriously affected. Hydrocorals of the genus *Millepora* spp. are among the organisms most affected by bleaching (Dias & Gondim, 2016). Particularly, *M. alcicornis* has been considered very vulnerable to temperature rise, since it has suffered severe bleaching episodes in different times and locations, such as in the Great Barrier Reef (Marshall & Baird, 2000), where an 85% mortality of this species was observed during the summer of 2005; in the Florida Keys reef in the summers of 2006 and 2007 (Wagner, Kramer & Van Woesik, 2010), and in Puerto Rico and the Caribbean during 1987, 1993, 1995, 1998, 2003, 2005, 2009, and 2010 (Dias & Gondim, 2016).

In the present study, normal and visibly bleached fragments of the “fire coral” *M. alcicornis*, (Figs. 1A and 1B) were collected in the Parque Nacional Arrecife de Puerto Morelos (Quintana Roo, México) in November 2016, the warmest year in NOAA’s 137-year series (NOAA, 2017). Since bleaching is provoked by a decrease in symbionts population and a reduction in the concentration of their photosynthetic pigments (Hoegh-Guldberg, 1999; Lesser, 2006, 2011; Obura, 2009), chlorophyll contents, and symbiont’s density were determined in *M. alcicornis*. As was expected, chlorophyll a and chlorophyll c2 concentrations per cm^2^ of hydrocoral were significantly lower in the bleached specimens (Fig. 1C), which indicates that warming provoked breakdown of the hydrocoral-algae symbiosis due to photoinhibition, in a similar way as that observed in Anthozoan species (Warner, Fitt & Schmidt, 1999; Takahashi & Murata, 2008; Lesser, 2011). Additionally, bleached *M. alcicornis* specimens showed a decrease of 40% in the density of symbionts per square centimeter (Fig. 1D), which corresponds to a “moderate bleaching” according to the categories used in the ReefBase (Oliver, Berkelmans & Eakin, 2009). Unlike what has been previously observed, the *M. alcicornis* colonies examined in the present study after the bleaching event of 2015–2016, did not show severe bleaching. This could indicate that this Caribbean hydrocoral is developing a certain degree of thermotolerance, which might be associated with the switching of symbionts, as reported in other species (Silverstein, Cunning & Baker, 2015; Bay et al., 2016; Swain et al., 2018). However, this hypothesis should be proven in future studies.

Comparative proteomic studies have been used to study the effect of heat stress on the total proteome from some Anthozoan species that include: *Acropora palmata*, *Acropora microphthalma*, *Pocillopora acuta, Seriatopora hystrix*, and *Aiptasia pallida*. The results derived from such investigations have shown that thermal stress, which is responsible for coral bleaching, provokes the differential expression of several proteins involved in different processes essential for the survival of the cnidarians, including cytoskeleton organization, thermal and UV stress response, redox state, immunity, calcium homeostasis, transcription factors, exocytosis, and metabolism (Weston et al., 2015; Ricaurte et al., 2016; Oakley et al., 2017; Mayfield et al., 2018a, 2018b). However, to date there are no reports about the effect of breakdown of the cnidarian-*Symbiodinium* symbiosis due to thermal stress on the expression of proteins, including toxins, in an organism of the class Hydrozoa. Therefore, in this study the differential expression of proteins in the soluble proteomes from normal and bleached *M. alcicornis* specimens was analyzed.

The extraction of high-quality proteins from corals with calcareous exoskeleton is not an easy task (Cheng et al., 2018). The most common methods reported for protein extraction include the use of trizol (Garcia et al., 2016; Mayfield et al., 2018a), glass bead-assisted extraction (Weston et al., 2015), and sonication-assisted extraction with rehydration buffer (Ricaurte et al., 2016). However, the method chosen for the extraction of *M. alcicornis* proteins involved osmotic shock in bidistilled water. This method causes the discharge of the nematocysts content (Ibarra-Alvarado et al., 2007; García-Arredondo et al., 2015, 2015, 2016; Morabito et al., 2012; Hernández-Matehuala et al., 2015).

Sodium dodecyl sulfate–polyacrylamide gel electrophoresis evidenced that soluble proteomes from normal and bleached *M. alcicornis* contain proteins with a broad range of molecular weights (Fig. 2). Most of the bands detected in both electrophoretic profiles are within the range of 10–200 kDa. A similar electrophoretic pattern was observed for the proteome of isolated nematocysts from *Hydra magnipapillata*, (a well-known member of the Hydrozoa class), in which most of the protein bands are in the range of 10–70 kDa (Balasubramanian et al., 2012). Some differences were observed in the SDS–PAGE profiles obtained from normal and bleached hydrocorals, indicating a differential protein expression between both types of specimens.

Moreover, 2DE-PAGE showed that the soluble proteomes from normal and bleached specimens of *M. alcicornis* resolved 58 and 75 proteins, respectively. It is important to mention that in the case of the present investigation, we examined the effect of elevated seawater temperature on the soluble proteome of *M. alcicornis*, unlike what has been done in previous studies carried out on Anthozoan species, in which the impact of thermal stress on the total proteomes from *Acropora palmata*, *A. microphthalma*, *Pocillopora acuta, Seriatopora hystrix*, and *Aiptasia pallida* was analyzed (Weston et al., 2015; Ricaurte et al., 2016; Oakley et al., 2017; Mayfield et al., 2018a, 2018b). After bleaching, the differential expression of 17 proteins was observed in *M. alcicornis*, six proteins were up-regulated, while 11 were down-regulated.

Calmodulin, actin, and collagen are some of the proteins differentially expressed in bleached specimens of *M. alcicornis.* Previous studies have reported the differential expression of these same proteins, which are involved in calcium homeostasis, cytoskeleton, and extracellular matrix (ECM) in Anthozoan species subjected to bleaching (Bay & Palumbi, 2015; Pinzón et al., 2015; Raina et al., 2015; Seneca & Palumbi, 2015; Weston et al., 2015; Maor-Landaw & Levy, 2016; Oakley et al., 2016, 2017; Ricaurte et al., 2016; Sloan & Sawyer, 2016; Huang et al., 2017; Ruiz-Jones & Palumbi, 2017; Traylor-Knowles et al., 2017; Mayfield et al., 2018a).

Calmodulin is a key Ca^2+^ sensor, whose signaling is important in numerous cellular processes, such as cell cycle and calcium homeostasis (Haeseleer et al., 2002; Desalvo et al., 2008; Reyes-Bermudez, Miller & Sprungala, 2012). Several studies have demonstrated that expression of genes and proteins involved in Ca^2+^ homeostasis is modified after bleaching in *Acropora palmata*, *Orbicella faveolata*, *Acropora millepora*, and *Galaxea astreata* (Desalvo et al., 2008, 2010; Rodriguez-Lanetty, Harii & Hoegh-Guldberg, 2009; Moya et al., 2012; Ricaurte et al., 2016; Huang et al., 2018). In the case of *M. alcicornis*, CaM was up-regulated after bleaching, which implies that changes are occurring in biomineralization and other calcium-dependent processes related to the development of the hydrocorals. It has been shown that CaM overexpression and a rupture in Ca^2+^ homeostasis are linked to an abnormal reorganization of the actin cytoskeleton (Desalvo et al., 2008; Reyes-Bermudez, Miller & Sprungala, 2012).

Actin was another protein that was up-regulated in bleached *M. alcicornis* specimens, in a similar way to what was found in scleractinian corals such as *Acropora palmata, Stylophora pistillata*, and *Montastraea faveolata* (Desalvo et al., 2008; Kenkel, Meyer & Matz, 2013; Maor-Landaw et al., 2014; Ricaurte et al., 2016; Louis et al., 2017). Actin is implicated in the construction of filaments and supports the majority of motile events in eukaryotic cells (Fletcher & Mullins, 2010). The increase in the expression of actin observed in bleached hydrocorals might be related to the relocation of symbionts as a mechanism of photoprotection (Petrou, Ralph & Nielsen, 2017) and to the increase of heterotrophic feeding to mitigate the energetic imbalance by the departure of symbionts (Wooldridge, 2014; Tremblay et al., 2016), which requires a significant contribution by the cytoskeleton to collect and transport nutrients.

Among the proteins that showed a down-regulation in bleached hydrocorals was collagen. This result is in agreement with what was found in specimens of *Acropora palmata, Stylophora pistillata*, and *Anemonia viridis* that were subjected to bleaching conditions (Moya et al., 2012; Maor-Landaw & Levy, 2016; Ricaurte et al., 2016). Collagen, abundantly found in the calicoblastic space, is central during the biomineralization process since it provides the structural support to which proteins of the subfamily CARP and analog proteins bind in nucleation and mineral growth sites (Mohamed et al., 2016). Moreover, a decline in symbiont´s density has been related to changes in the active volume of calcification due to a reduction in dissolved inorganic carbon (Drake et al., 2013; D’Olivo & McCulloch, 2017). Thus, down-regulation of collagen and reduced symbiont´s density suggest that thermal stress induces a decrease in the rate of calcification in the hydrocoral *M. alcicornis*.

The exocytosis multiprotein complex provides spatial targeting of exocytotic vesicles to the plasma membrane (Picco et al., 2017), and therefore is related to the process of symbionts expulsion (Bieri et al., 2016). In fact, the exocytosis complex component 4 was up-regulated in *Porites astreoides*, and has been proposed as a biomarker of coral thermal stress (Kenkel, Meyer & Matz, 2013; Kenkel et al., 2014). In contrast, exocytosis complex component 3 was down-regulated in bleached *M. alcicornis*, as previously reported for other Endo-exo phagocytosis-related proteins in *Acropora palmata* (Ricaurte et al., 2016). Thus, the proposal that up-regulation of proteins that are part of the exocytosis complex represents a biomarker of thermal stress is still controversial.

Three of the soluble proteins from *M. alcicornis*, which were differentially expressed in bleached hydrocorals, showed amino acid sequence similarity to potential toxins. Despite the ecological relevance of reef forming hydrocorals, the information regarding the chemical structure and mechanism of action of their toxins is scarce. Previous studies carried out by our research group showed that the aqueous extract of *M. alcicornis* caused hemolysis of rat erythrocytes and showed PLA2 activity (Hernández-Matehuala et al., 2015). Zymographic analysis of this extract revealed that it contained ∼28–30 kDa cytolysins with PLA2 activity; ∼200 kDa cytoloysins, which do not have PLA2 activity (Hernández-Matehuala et al., 2015) and proteins with proteolytic activity at ∼25, ∼40, and 80–200 kDa (Olguín-López Norma, Hernández-Elizarraga Víctor, Hernández-Matehuala Rosalina, Cruz-Hernández Andrés, Guevara-González Ramón, Caballero-Perez Juan, Ibarra-Alvarado César , and Rojas-Molina Alejandra, 2018, unpublished data). Interestingly, we found that bleaching induced up-regulation of proteins that showed sequence homology with DELTA-actitoxin-Ate1a-like protein from the sea anemone *Exaiptasia pallida* (Sunagawa et al., 2009) and a putative PLA2 from *Hydra magnipapillata* (Sher et al., 2005). In contrast, bleaching elicited down-regulation of a protein that has sequence similarity with a ATP-dependent Zinc metalloprotease from *Aiptasia pallida* (Sunagawa et al., 2009).

Cnidarian toxins are mainly proteins and peptides (Anderluh & Maček, 2002; Nevalainen et al., 2004; Mariottini et al., 2015; Podobnik & Anderluh, 2017) and can be classified into three categories based on their mechanism of action: enzymes (PLA2 and metalloproteinases); pore-forming proteins (actinoporins, jellyfish toxins, hydralysins-related toxins, and membrane attack complex-perforins), and neurotoxins (Jouiaei et al., 2015b). The most studied pore forming toxins of cnidarians are actinoporins, that have been found in many species of marine anemones (Anderluh & Maček, 2002; Kristan et al., 2009; Šuput, 2009). One of the proteins whose expression was up-regulated after bleaching displayed sequence similarity with actinoporin DELTA-actitoxin-Ate1a-like protein. This finding suggests that *M. alcicornis* produce PFTs, whose structure might be related to that of the anemone actinoporins, which is interesting considering that this type of toxins have been found in only one species of the class Hydrozoa, *Hydra magnipapillata* (Jouiaei et al., 2015b).

On the other hand, the expression of a protein that exhibited sequence similarity to a putative PLA2 of 15.7 kDa from *Hydra magnipapillata*, which was found by a large-scale search at the *Hydra magnipapillata* EST Database, was up-regulated after bleaching. This Hydra PLA2 showed homology with PLA2s from *Apis mellifera*, the lizard *Heloderma uspectum* (“Gila Monster”), and the scorpions *Mesobuthus tamulus* and *Pandinus imperator*, mainly in the calcium binding domain and in the catalytic site. All of these PLA2s display neurotoxic activity and belong to the secreted PLA2 (sPLA2) group III (sPLA2-III) (Sher et al., 2005). In contrast to snake venom sPLA2s (Costa, Camargo & Antunes, 2015; Kordiš & Križaj, 2017; Tonello & Rigoni, 2017), cnidarian sPLA2s have been poorly characterized and their enzymatic action greatly differs between species (Nevalainen et al., 2004).

The present study also evidenced that proteins that displayed sequence similarity to disintegrin and metalloproteinase domain-containing protein 7 and an ATP-dependent Zinc metalloprotease showed down-regulation after bleaching. Metalloproteinases have been detected in some cnidarian nematocyst venoms, such as those of *Stomolophus meleagris* (Li et al., 2014), *Olindias sambaquiensis* (Knittel et al., 2016), *Pelagia noctiluca* (Frazão et al., 2017), and *Chironex fleckeri* (Jouiaei et al., 2015a). This class of toxins shows a great diversity of effects. Snake venom metalloproteinases (SVMPs) affect the ECM in various ways, including the release of ECM-derived biologically-active peptides, which exerts either reparing or damaging effects on tissues (Gutiérrez et al., 2016). These enzymes are also involved in the proteolytic processing of other venom proteins (Da Silveira et al., 2007) and are capable of causing severe inflammation by disrupting capillary vessels and tissues (Weston et al., 2013). However, the different kinds of cnidarian metalloproteinases and their effects are not yet clear. It has been demonstrated that SVMPs undergo various post-translational modifications, which contribute to their great functional diversity (Moura-da-Silva et al., 2016). This could be the case of cnidarian metalloproteinases. If this is so, then it could be hypothesized that the imbalance of energy produced by symbionts expulsion or death induce cnidarians to optimize their energy expenditure, which impairs the metalloproteases synthesis that represents a great energy investment.

Cnidarians employ toxins as a defense against predators and in prey capture to obtain heterotrophic nutrition (Anderluh & Maček, 2002; Anderluh et al., 2011; Houlbrèque, Rodolfo-Metalpa & Ferrier-Pagès, 2015; Ben-Ari, Paz & Sher, 2018). Particularly, both, PFTs and PLA2s produce cytolysis and play a very important role in defensive and offensive actions, since they provoke the destruction of cell membranes (Rojko et al., 2016; Macrander & Daly, 2016; Podobnik & Anderluh, 2017). It has been documented that certain corals, such as *Oculina arbusculata*, can increase their heterotrophic nutrition to maintain and restore energy reserves after a bleaching event (Grottoli, Rodrigues & Palardy, 2006; Aichelman et al., 2016). In the case of the present study, the differential expression of potential toxins suggests that thermal stress alters heterotrophic competences to mitigate the energy imbalance in hydrocorals. However, heat stress did not affect the hemolytic, PLA2, and proteolytic activity of *M. alcicornis* soluble proteome, which suggests that the cytolysis induced by the toxins of this hydrocoral is mainly produced by enzymes and PFTs (Nevalainen et al., 2004; Lee et al., 2011; Anderluh et al., 2011; Jouiaei et al., 2015a; Rojko et al., 2016; Knittel et al., 2016), whose expression was not affected after suffering moderate bleaching. It is worth mentioning that in a previous study carried out on *M. alcicornis* specimens collected in the Mexican Caribbean in 2008, we found that thermal stress did not significantly modify the PLA2 activity of the aqueous extract prepared from bleached *M. alcicornis* (García-Arredondo et al., 2015), which agrees with what we found in the present study with specimens collected in 2016. These findings seem to indicate that *M. alcicornis* subjected to moderate bleaching does not lose its capability to synthesize cytolysins. Evidently, it is important to continue investigating the impact of elevated ocean temperatures that provoke bleaching on the synthesis of toxins produced by reef forming cnidarians and the significance of heterotrophic feeding as a mechanism to counteract the deleterious effects of this phenomenon.

## Conclusions

The present study represents the first report of the effect of thermal stress on the proteomic profile of a reef forming cnidarian of the class Hydrozoa. The decrease in symbiont’s density and chlorophyll a and c2 levels suggests that ENSO 2015–2016 induced a moderate bleaching in colonies of *M. alcicornis* that inhabit the Mexican Caribbean. Proteins involved in various important cellular processes, such as calcium homeostasis, exocytosis, and cytoskeleton organization were differentially expressed in bleached hydrocorals. Three of the differentially expressed proteins showed amino acid sequence similarity to potential toxins, however, the hemolytic, PLA2, and proteolytic activity of bleached *M. alcicornis* soluble proteome was not modified. These results suggests that the cytolytic effect induced by this hydrocoral is produced by toxins whose synthesis is not altered after bleaching, which allow us to hypothesize that *M. alcicornis* is able to prey heterotrophically when suffering from moderate bleaching, giving it a better chance to face the effects of high temperature.

## Supplemental Information

10.7717/peerj.6593/supp-1 Supplemental Information 1 Raw data for Figure 1 and Table 2.

## Additional Information and Declarations

### Field Study Permissions

The following information was supplied relating to field study approvals (i.e., approving body and any reference numbers): A collection permit was issued by Secretaría de Agricultura, Desarrollo Rural, Pesca y Alimentación (SAGARPA) (permit number PPF/DGOPA-139/15).

### Data Availability

The following information was supplied regarding data availability: The raw data are available in the Supplemental Files.

## References

[ref-1] Aichelman HE, Townsend JE, Courtney TA, Baumann JH, Davies SW, Castillo KD (2016). Heterotrophy mitigates the response of the temperate coral *Oculina arbuscula* to temperature stress. Ecology and Evolution.

[ref-2] Anderluh G, Maček P (2002). Cytolytic peptide and protein toxins from sea anemones (Anthozoa: Actiniaria). Toxicon.

[ref-3] Anderluh G, Sepčić K, Turk T, Maček P (2011). Cytolytic proteins from cnidarians—an overview. Acta Chimica Slovenica.

[ref-4] Anthony KR (2016). Coral reefs under climate change and ocean acidification: challenges and opportunities for management and policy. Annual Review of Environment and Resources.

[ref-5] Baird AH, Bhagooli R, Ralph PJ, Takahashi S (2009). Coral bleaching: the role of the host. Trends in Ecology & Evolution.

[ref-6] Baker AC, Glynn PW, Riegl B (2008). Climate change and coral reef bleaching: an ecological assessment of long-term impacts, recovery trends and future outlook. Estuarine, Coastal and Shelf Science.

[ref-7] Balasubramanian PG, Beckmann A, Warnken U, Schnölzer M, Schüler A, Bornberg-Bauer E, Holstein TW, Özbek S (2012). Proteome of hydra nematocyst. Journal of Biological Chemistry.

[ref-9] Bay LK, Doyle J, Logan M, Berkelmans R (2016). Recovery from bleaching is mediated by threshold densities of background thermo-tolerant symbiont types in a reef-building coral. Royal Society Open Science.

[ref-10] Bay RA, Palumbi SR (2015). Rapid acclimation ability mediated by transcriptome changes in reef-building corals. Genome Biology and Evolution.

[ref-11] Ben-Ari H, Paz M, Sher D (2018). The chemical armament of reef-building corals: inter- and intra-specific variation and the identification of an unusual actinoporin in *Stylophora pistilata*. Scientific Reports.

[ref-12] Bieri T, Onishi M, Xiang T, Grossman AR, Pringle JR (2016). Relative contributions of various cellular mechanisms to loss of algae during cnidarian bleaching. PLOS ONE.

[ref-13] Bonesso JL, Leggat W, Ainsworth TD (2017). Exposure to elevated sea-surface temperatures below the bleaching threshold impairs coral recovery and regeneration following injury. PeerJ.

[ref-14] Cheng H, Zhao H, Yang T, Ruan S, Wang H, Xiang N, Zhou H, Li QX, Diao X (2018). Comparative evaluation of five protocols for protein extraction from stony corals (Scleractinia) for proteomics. Electrophoresis.

[ref-15] Costa SK, Camargo EA, Antunes E (2015). Inflammatory Action of Secretory PLA2 from Snake Venoms. Toxins and Drug Discovery.

[ref-16] Cragg GM, Newman DJ (2013). Natural products: a continuing source of novel drug leads. Biochimica et Biophysica Acta (BBA)—General Subjects.

[ref-17] Cramer KL, O’Dea A, Clark TR, Zhao J, Norris RD (2017). Prehistorical and historical declines in Caribbean coral reef accretion rates driven by loss of parrotfish. Nature Communications.

[ref-18] Da Silveira RB, Wille AC, Chaim OM, Appel MH, Silva DT, Franco CR, Toma L, Mangili OC, Gremski W, Dietrich CP, Nader HB, Veiga SS (2007). Identification, cloning, expression and functional characterization of an astacin-like metalloprotease toxin from *Loxosceles intermedia* (brown spider) venom. Biochemical Journal.

[ref-19] Davy SK, Allemand D, Weis VM (2012). Cell biology of cnidarian-dinoflagellate symbiosis. Microbiology and Molecular Biology Reviews.

[ref-20] Desalvo MK, Sunagawa S, Voolstra CR, Medina M (2010). Transcriptomic responses to heat stress and bleaching in the elkhorn coral *Acropora palmata*. Marine Ecology Progress Series.

[ref-21] Desalvo MK, Voolstra CR, Sunagawa S, Schwarz JA, Stillman JH, Coffroth MA, Szmant AM, Medina M (2008). Differential gene expression during thermal stress and bleaching in the Caribbean coral *Montastraea faveolata*. Molecular Ecology.

[ref-22] Dias TLP, Gondim AI (2016). Bleaching in scleractinians, hydrocorals, and octocorals during thermal stress in a northeastern Brazilian reef. Marine Biodiversity.

[ref-23] D’Olivo JP, McCulloch MT (2017). Response of coral calcification and calcifying fluid composition to thermally induced bleaching stress. Scientific Reports.

[ref-24] Douglas AE (2003). Coral bleaching—how and why?. Marine Pollution Bulletin.

[ref-25] Drake JL, Mass T, Haramaty L, Zelzion E, Bhattacharya D, Falkowski PG (2013). Proteomic analysis of skeletal organic matrix from the stony coral *Stylophora pistillata*. Proceedings of the National Academy of Sciences of the United States of America.

[ref-27] Eakin CM, Liu G, Gomez AM, La JLD (2017). Ding, dong, the witch is dead (?)—three years of global coral bleaching 2014–2017. Reef Encounter.

[ref-28] Eakin CM, Morgan JA, Heron SF, Smith TB, Liu G, Alvarez-Filip L, Baca B, Bartels E, Bastidas C, Bouchon C, Brandt M, Bruckner AW, Bunkley-Williams L, Cameron A, Causey BD, Chiappone M, Christensen TRL, Crabbe MJC, Day O, De la Guardia E, Díaz-Pulido G, DiResta D, Gil-Agudelo DL, Gilliam DS, Ginsburg RN, Gore S, Guzmán HM, Hendee JC, Hernández-Delgado EA, Husain E, Jeffrey CFG, Jones RJ, Jordán-Dahlgren E, Kaufman LS, Kline DI, Kramer PA, Lang JC, Lirman D, Mallela J, Manfrino C, Maréchal J-P, Marks K, Mihaly J, Jeff Miller W, Mueller EM, Muller EM, Orozco Toro CA, Oxenford HA, Ponce-Taylor D, Quinn N, Ritchie KB, Rodríguez S, Rodríguez Ramírez A, Romano S, Samhouri JF, Sánchez JA, Schmahl GP, Shank BV, Skirving WJ, Steiner SCC, Villamizar E, Walsh SM, Walter C, Weil E, Williams EH, Roberson KW, Yusuf Y (2010). Caribbean corals in crisis: record thermal stress, bleaching, and mortality in 2005. PLOS ONE.

[ref-29] Fletcher DA, Mullins RD (2010). Cell mechanics and the cytoskeleton. Nature.

[ref-30] Fransolet D, Roberty S, Plumier J-C (2012). Establishment of endosymbiosis: the case of cnidarians and *Symbiodinium*. Journal of Experimental Marine Biology and Ecology.

[ref-31] Frazão B, Campos A, Osório H, Thomas B, Leandro S, Teixeira A, Vasconcelos V, Antunes A (2017). Analysis of *Pelagia noctiluca* proteome reveals a red fluorescent protein, a zinc metalloproteinase and a peroxiredoxin. Protein Journal.

[ref-32] Garcia GD, Santos EO, Sousa GV, Zingali RB, Thompson CC, Thompson FL (2016). Metaproteomics reveals metabolic transitions between healthy and diseased stony coral *Mussismilia braziliensis*. Molecular Ecology.

[ref-33] García-Arredondo A, Rojas-Molina A, Bah M, Ibarra-Alvarado C, Gallegos-Corona MA, García-Servín M (2015). Systemic toxic effects induced by the aqueous extract of the fire coral *Millepora complanata* and partial purification of thermostable neurotoxins with lethal effects in mice. Comparative Biochemistry and Physiology Part C: Toxicology & Pharmacology.

[ref-34] García-Arredondo A, Rojas-Molina A, Bah M, Ibarra-Alvarado C, Gallegos-Corona MA, García-Servín M (2015). Systemic toxic effects induced by the aqueous extract of the fire coral Millepora complanata and partial purification of thermostable neurotoxins with lethal effects in mice. Comparative Biochemistry and Physiology Part C: Toxicology & Pharmacology.

[ref-35] García-Arredondo A, Rojas-Molina A, Ibarra-Alvarado C, Lazcano-Pérez F, Arreguín-Espinosa R, Sánchez-Rodríguez J (2016). Composition and biological activities of the aqueous extracts of three scleractinian corals from the Mexican Caribbean: *Pseudodiploria strigosa*, *Porites astreoides* and *Siderastrea siderea*. Journal of Venomous Animals and Toxins including Tropical Diseases.

[ref-37] Grottoli AG, Rodrigues LJ, Palardy JE (2006). Heterotrophic plasticity and resilience in bleached corals. Nature.

[ref-38] Grottoli AG, Warner ME, Levas SJ, Aschaffenburg MD, Schoepf V, McGinley M, Baumann J, Matsui Y (2014). The cumulative impact of annual coral bleaching can turn some coral species winners into losers. Global Change Biology.

[ref-39] Gutiérrez JM, Escalante T, Rucavado A, Herrera C, Fox JW (2016). A comprehensive view of the structural and functional alterations of extracellular matrix by snake venom metalloproteinases (SVMPs): novel perspectives on the pathophysiology of envenoming. Toxins.

[ref-40] Haeseleer F, Imanishi Y, Sokal I, Filipek S, Palczewski K (2002). Calcium-binding proteins: intracellular sensors from the calmodulin superfamily. Biochemical and Biophysical Research Communications.

[ref-41] Hernández-Matehuala R, Rojas-Molina A, Vuelvas-Solórzano AA, Garcia-Arredondo A, Alvarado CI, Olguín-López N, Aguilar M (2015). Cytolytic and systemic toxic effects induced by the aqueous extract of the fire coral *Millepora alcicornis* collected in the Mexican Caribbean and detection of two types of cytolisins. Journal of Venomous Animals and Toxins including Tropical Diseases.

[ref-42] Heron SF, Maynard JA, Van Hooidonk R, Eakin CM (2016). Warming trends and bleaching stress of the world’s coral reefs 1985–2012. Scientific Reports.

[ref-43] Hillyer KE, Dias D, Lutz A, Roessner U, Davy SK (2018). 13C metabolomics reveals widespread change in carbon fate during coral bleaching. Metabolomics.

[ref-44] Hillyer KE, Dias DA, Lutz A, Wilkinson SP, Roessner U, Davy SK (2017). Metabolite profiling of symbiont and host during thermal stress and bleaching in the coral *Acropora aspera*. Coral Reefs.

[ref-45] Hoegh-Guldberg O (1999). Climate change, coral bleaching and the future of the world’s coral reefs. Marine and Freshwater Research.

[ref-46] Hoegh-Guldberg O, Bruno JF (2010). The impact of climate change on the world’s marine ecosystems. Science.

[ref-47] Houlbreque F, Ferrier-Pagès C (2009). Heterotrophy in tropical scleractinian corals. Biological Reviews.

[ref-48] Houlbrèque F, Rodolfo-Metalpa R, Ferrier-Pagès C, Woodley CM, Downs CA, Bruckner AW, Porter JW, Galloway SB (2015). Heterotrophic nutrition of tropical, temperate and deep-sea corals. Diseases of Coral.

[ref-49] Huang C, Morlighem J-ÉR, Cai J, Liao Q, Perez CD, Gomes PB, Guo M, Rádis-Baptista G, Lee SM-Y (2017). Identification of long non-coding RNAs in two anthozoan species and their possible implications for coral bleaching. Scientific Reports.

[ref-50] Huang Y, Yuan J, Zhang Y, Peng H, Liu L (2018). Molecular cloning and characterization of calmodulin-like protein CaLP from the Scleractinian coral *Galaxea astreata*. Cell Stress and Chaperones.

[ref-51] Hughes TP, Anderson KD, Connolly SR, Heron SF, Kerry JT, Lough JM, Baird AH, Baum JK, Berumen ML, Bridge TC, Claar DC, Eakin CM, Gilmour JP, Graham NAJ, Harrison H, Hobbs J-PA, Hoey AS, Hoogenboom M, Lowe RJ, McCulloch MT, Pandolfi JM, Pratchett M, Schoepf V, Torda G, Wilson SK (2018). Spatial and temporal patterns of mass bleaching of corals in the Anthropocene. Science.

[ref-52] Hughes TP, Barnes ML, Bellwood DR, Cinner JE, Cumming GS, Jackson JBC, Kleypas J, Van De Leemput IA, Lough JM, Morrison TH, Palumbi SR, Van Nes EH, Scheffer M (2017a). Coral reefs in the Anthropocene. Nature.

[ref-53] Hughes TP, Kerry JT, Álvarez-Noriega M, Álvarez-Romero JG, Anderson KD, Baird AH, Babcock RC, Beger M, Bellwood DR, Berkelmans R, Bridge TC, Butler IR, Byrne M, Cantin NE, Comeau S, Connolly SR, Cumming GS, Dalton SJ, Diaz-Pulido G, Eakin CM, Figueira WF, Gilmour JP, Harrison HB, Heron SF, Hoey AS, Hobbs J-PA, Hoogenboom MO, Kennedy EV, Kuo C, Lough JM, Lowe RJ, Liu G, McCulloch MT, Malcolm HA, McWilliam MJ, Pandolfi JM, Pears RJ, Pratchett MS, Schoepf V, Simpson T, Skirving WJ, Sommer B, Torda G, Wachenfeld DR, Willis BL, Wilson SK (2017b). Global warming and recurrent mass bleaching of corals. Nature.

[ref-54] Ibarra-Alvarado C, García JA, Aguilar MB, Rojas A, Falcón A, De la Cotera EPH (2007). Biochemical and pharmacological characterization of toxins obtained from the fire coral *Millepora complanata*. Comparative Biochemistry and Physiology Part C: Toxicology & Pharmacology.

[ref-55] Jackson JBC, Donovan MK, Cramer KL, Lam VV (2014). Status and Trends of Caribbean Coral Reefs: 1970-2012.

[ref-56] Jain R, Sonawane S, Mandrekar N (2008). Marine organisms: potential source for drug discovery. Current Science.

[ref-57] Jeffrey SW, Humphrey GF (1975). New spectrophotometric equations for determining chlorophylls a, b, c1 and c2 in higher plants, algae and natural phytoplankton. Biochemie und Physiologie der Pflanzen.

[ref-58] Jouiaei M, Casewell NR, Yanagihara AA, Nouwens A, Cribb BW, Whitehead D, Jackson TNW, Ali SA, Wagstaff SC, Koludarov I, Alewood P, Hansen J, Fry BG (2015a). Firing the sting: chemically induced discharge of cnidae reveals novel proteins and peptides from box jellyfish (*Chironex fleckeri*) venom. Toxins.

[ref-59] Jouiaei M, Yanagihara AA, Madio B, Nevalainen TJ, Alewood PF, Fry BG (2015b). Ancient venom systems: a review on cnidaria toxins. Toxins.

[ref-60] Kenkel CD, Meyer E, Matz MV (2013). Gene expression under chronic heat stress in populations of the mustard hill coral (*Porites astreoides*) from different thermal environments. Molecular Ecology.

[ref-61] Kenkel CD, Sheridan C, Leal MC, Bhagooli R, Castillo KD, Kurata N, McGinty E, Goulet TL, Matz MV (2014). Diagnostic gene expression biomarkers of coral thermal stress. Molecular Ecology Resources.

[ref-62] Knittel PS, Long PF, Brammall L, Marques AC, Almeida MT, Padilla G, Moura-da-Silva AM (2016). Characterising the enzymatic profile of crude tentacle extracts from the South Atlantic jellyfish *Olindias sambaquiensis* (Cnidaria: Hydrozoa). Toxicon.

[ref-63] Kordiš D, Križaj I, Gopalakrishnakone P, Inagaki H, Vogel CW, Mukherjee AK, Rahmy TR (2017). Secreted Phospholipases A2 with β-Neurotoxic Activity. Snake Venoms.

[ref-64] Kristan KČ, Viero G, Dalla Serra M, Maček P, Anderluh G (2009). Molecular mechanism of pore formation by actinoporins. Toxicon.

[ref-67] Mariottini G, Bonello G, Giacco E, Pane L (2015). Neurotoxic and neuroactive compounds from Cnidaria: five decades of research central nervous system agents in medicinal chemistry (formerly current medicinal chemistry-central nervous system agents). Central Nervous System Agents in Medicinal Chemistry.

[ref-68] Lee H, Jung E, Kang C, Yoon WD, Kim J-S, Kim E (2011). Scyphozoan jellyfish venom metalloproteinases and their role in the cytotoxicity. Toxicon: Official Journal of the International Society on Toxinology.

[ref-69] Lesser MP (2006). Oxidative stress in marine environments: biochemistry and physiological ecology. Annual Review of Physiology.

[ref-70] Lesser MP (2007). Coral reef bleaching and global climate change: can corals survive the next century?. Proceedings of the National Academy of Sciences of the United States of America.

[ref-71] Lesser MP, Dubinsky Z, Stambler N (2011). Coral bleaching: causes and mechanisms. Coral Reefs: An Ecosystem in Transition.

[ref-72] Levas S, Grottoli AG, Schoepf V, Aschaffenburg M, Baumann J, Bauer JE, Warner ME (2016). Can heterotrophic uptake of dissolved organic carbon and zooplankton mitigate carbon budget deficits in annually bleached corals?. Coral Reefs.

[ref-73] Lewis JB (2006). Biology and ecology of the hydrocoral *Millepora* on coral reefs. Advances in Marine Biology.

[ref-74] Li T, Ding T, Li J (2017). Medicinal purposes: bioactive metabolites from marine-derived organisms. Mini Reviews in Medicinal Chemistry.

[ref-75] Li R, Yu H, Xue W, Yue Y, Liu S, Xing R, Li P (2014). Jellyfish venomics and venom gland transcriptomics analysis of *Stomolophus meleagris* to reveal the toxins associated with sting. Journal of Proteomics.

[ref-76] Lough JM (2016). Coral reefs: turning back time. Nature.

[ref-77] Louis YD, Bhagooli R, Kenkel CD, Baker AC, Dyall SD (2017). Gene expression biomarkers of heat stress in scleractinian corals: promises and limitations. Comparative Biochemistry and Physiology Part C: Toxicology & Pharmacology.

[ref-78] Macrander J, Daly M (2016). Evolution of the cytolytic pore-forming proteins (Actinoporins) in sea anemones. Toxins.

[ref-79] Maor-Landaw K, Karako-Lampert S, Ben-Asher HW, Goffredo S, Falini G, Dubinsky Z, Levy O (2014). Gene expression profiles during short-term heat stress in the red sea coral *Stylophora pistillata*. Global Change Biology.

[ref-80] Maor-Landaw K, Levy O (2016). Gene expression profiles during short-term heat stress; branching vs. massive Scleractinian corals of the Red Sea. PeerJ.

[ref-81] Marshall PA, Baird AH (2000). Bleaching of corals on the Great Barrier Reef: differential susceptibilities among taxa. Coral Reefs.

[ref-82] Mayer AM, Nguyen M, Newman DJ, Glaser KB (2016). The marine pharmacology and pharmaceuticals pipeline in 2015. FASEB Journal.

[ref-83] Mayfield AB, Chen Y-J, Lu C-Y, Chen C-S (2018a). The proteomic response of the reef coral *Pocillopora acuta* to experimentally elevated temperatures. PLOS ONE.

[ref-84] Mayfield AB, Chen Y-J, Lu C-Y, Chen C-S (2018b). Exploring the environmental physiology of the Indo-Pacific Reef Coral Seriatopora hystrix with differential proteomics. Open Journal of Marine Science.

[ref-85] Mohamed AR, Cumbo V, Harii S, Shinzato C, Chan CX, Ragan MA, Bourne DG, Willis BL, Ball EE, Satoh N, Miller DJ (2016). The transcriptomic response of the coral *Acropora digitifera* to a competent *Symbiodinium* strain: the symbiosome as an arrested early phagosome. Molecular Ecology.

[ref-86] Mora C, Graham NAJ, Nyström M (2016). Ecological limitations to the resilience of coral reefs. Coral Reefs.

[ref-87] Morabito R, Condello S, Currò M, Marino A, Ientile R, La Spada G (2012). Oxidative stress induced by crude venom from the jellyfish *Pelagia noctiluca* in neuronal-like differentiated SH-SY5Y cells. Toxicology in Vitro.

[ref-88] Moura-da-Silva AM, Almeida MT, Portes-Junior JA, Nicolau CA, Gomes-Neto F, Valente RH (2016). Processing of snake venom metalloproteinases: generation of toxin diversity and enzyme inactivation. Toxins.

[ref-89] Moya A, Ganot P, Furla P, Sabourault C (2012). The transcriptomic response to thermal stress is immediate, transient and potentiated by ultraviolet radiation in the sea anemone *Anemonia viridis*. Molecular Ecology.

[ref-90] Mumby PJ, Van Woesik R (2014). Consequences of ecological, evolutionary and biogeochemical uncertainty for coral reef responses to climatic stress. Current Biology.

[ref-91] Nagaraju S, Mahadeswaraswamy YH, Girish KS, Kemparaju K (2006). Venom from spiders of the genus *Hippasa*: biochemical and pharmacological studies. Comparative Biochemistry and Physiology Part C: Toxicology & Pharmacology.

[ref-92] Neal BP, Khen A, Treibitz T, Beijbom O, O’Connor G, Coffroth MA, Knowlton N, Kriegman D, Mitchell BG, Kline DI (2017). Caribbean massive corals not recovering from repeated thermal stress events during 2005–2013. Ecology and Evolution.

[ref-93] Nevalainen TJ, Peuravuori HJ, Quinn RJ, Llewellyn LE, Benzie JA, Fenner PJ, Winkel KD (2004). Phospholipase A2 in cnidaria. Comparative Biochemistry and Physiology Part B: Biochemistry and Molecular Biology.

[ref-36] NOAA (2017). Global Climate Report—Annual 2016. https://www.ncdc.noaa.gov/sotc/global/201613.

[ref-94] Oakley CA, Ameismeier MF, Peng L, Weis VM, Grossman AR, Davy SK (2016). Symbiosis induces widespread changes in the proteome of the model cnidarian *Aiptasia*. Cellular Microbiology.

[ref-95] Oakley CA, Durand E, Wilkinson SP, Peng L, Weis VM, Grossman AR, Davy SK (2017). Thermal shock induces host proteostasis disruption and endoplasmic reticulum stress in the model symbiotic cnidarian *Aiptasia*. Journal of Proteome Research.

[ref-96] Obura DO (2009). Reef corals bleach to resist stress. Marine Pollution Bulletin.

[ref-97] Okazaki RR, Towle EK, Van Hooidonk R, Mor C, Winter RN, Piggot AM, Cunning R, Baker AC, Klaus JS, Swart PK, Langdon C (2016). Species-specific responses to climate change and community composition determine future calcification rates of Florida Keys reefs. Global Change Biology.

[ref-98] Oliver JK, Berkelmans R, Eakin CM, van Oppen MJH, Lough JM (2009). Coral Bleaching in Space and Time. Coral Bleaching. Ecological Studies.

[ref-101] Petrou K, Ralph PJ, Nielsen DA (2017). A novel mechanism for host-mediated photoprotection in endosymbiotic foraminifera. ISME Journal.

[ref-102] Picco A, Irastorza-Azcarate I, Specht T, Böke D, Pazos I, Rivier-Cordey A-S, Devos DP, Kaksonen M, Gallego O (2017). The in vivo architecture of the exocyst provides structural basis for exocytosis. Cell.

[ref-103] Pinzón JH, Kamel B, Burge CA, Harvell CD, Medina M, Weil E, Mydlarz LD (2015). Whole transcriptome analysis reveals changes in expression of immune-related genes during and after bleaching in a reef-building coral. Royal Society Open Science.

[ref-104] Podobnik M, Anderluh G (2017). Pore-forming toxins in Cnidaria. Seminars in Cell & Developmental Biology.

[ref-105] Raina JB, Lutz A, Motti CA, Miller DJ, Van Oppen MJH (2015). Host coenzyme Q redox state is an early biomarker of thermal stress in the coral *Acropora millepora*. PLOS ONE.

[ref-106] Reyes-Bermudez A, Miller DJ, Sprungala S (2012). The neuronal calcium sensor protein acrocalcin: a potential target of calmodulin regulation during development in the coral *Acropora millepora*. PLOS ONE.

[ref-107] Ricaurte M, Schizas NV, Ciborowski P, Boukli NM (2016). Proteomic analysis of bleached and unbleached *Acropora palmata*, a threatened coral species of the Caribbean. Marine Pollution Bulletin.

[ref-108] Rodriguez-Lanetty M, Harii S, Hoegh-Guldberg O (2009). Early molecular responses of coral larvae to hyperthermal stress. Molecular Ecology.

[ref-109] Roff G, Zhao J, Mumby PJ (2015). Decadal-scale rates of reef erosion following El Niño-related mass coral mortality. Global Change Biology.

[ref-110] Rojas-Molina A, García-Arredondo A, Ibarra-Alvarado C, Bah M (2012). *Millepora* (“fire corals”) species: toxinological studies until 2011. Advances in Environmental Research.

[ref-111] Rojko N, Dalla Serra M, Maček P, Anderluh G (2016). Pore formation by actinoporins, cytolysins from sea anemones. Biochimica et Biophysica Acta (BBA)—Biomembranes.

[ref-112] Ruiz-Jones LJ, Palumbi SR (2017). Tidal heat pulses on a reef trigger a fine-tuned transcriptional response in corals to maintain homeostasis. Science Advances.

[ref-113] Ruiz-Ramos DV, Weil E, Schizas NV (2014). Morphological and genetic evaluation of the hydrocoral *Millepora* species complex in the Caribbean. Zoological Studies.

[ref-114] Schägger H, Von Jagow G (1987). Tricine-sodium dodecyl sulfate-polyacrylamide gel electrophoresis for the separation of proteins in the range from 1 to 100 kDa. Analytical Biochemistry.

[ref-115] Seneca FO, Palumbi SR (2015). The role of transcriptome resilience in resistance of corals to bleaching. Molecular Ecology.

[ref-116] Sher D, Knebel A, Bsor T, Nesher N, Tal T, Morgenstern D, Cohen E, Fishman Y, Zlotkin E (2005). Toxic polypeptides of the hydra—a bioinformatic approach to cnidarian allomones. Toxicon.

[ref-117] Shirur KP, Ramsby BD, Iglesias-Prieto R, Goulet TL (2014). Biochemical composition of Caribbean gorgonians: implications for gorgonian—*Symbiodinium* symbiosis and ecology. Journal of Experimental Marine Biology and Ecology.

[ref-118] Silverstein RN, Cunning R, Baker AC (2015). Change in algal symbiont communities after bleaching, not prior heat exposure, increases heat tolerance of reef corals. Global Change Biology.

[ref-119] Sloan R, Sawyer S (2016). The role of cellular signaling during bleaching in the sea anemone, Aiptasia pallida. Proceedings of the West Virginia Academy of Science.

[ref-120] Sunagawa S, Wilson EC, Thaler M, Smith ML, Caruso C, Pringle JR, Weis VM, Medina M, Schwarz JA (2009). Generation and analysis of transcriptomic resources for a model system on the rise: the sea anemone *Aiptasia pallida* and its dinoflagellate endosymbiont. BMC Genomics.

[ref-121] Šuput D (2009). In vivo effects of cnidarian toxins and venoms. Toxicon.

[ref-122] Swain TD, Westneat MW, Backman V, Marcelino LA (2018). Phylogenetic analysis of symbiont transmission mechanisms reveal evolutionary patterns in thermotolerance and host specificity that enhance bleaching resistance among vertically transmitted Symbiodinium. European Journal of Phycology.

[ref-123] Takahashi S, Murata N (2008). How do environmental stresses accelerate photoinhibition?. Trends in Plant Science.

[ref-125] Tonello F, Rigoni M, Gopalakrishnakone P, Inagaki H, Vogel CW, Mukherjee A, Rahmy T (2017). Cellular Mechanisms of Action of Snake Phospholipase A2 Toxins. Snake Venoms. Toxinology.

[ref-126] Traylor-Knowles N, Rose NH, Sheets EA, Palumbi SR (2017). Early transcriptional responses during heat stress in the coral *Acropora hyacinthus*. Biological Bulletin.

[ref-127] Tremblay P, Gori A, Maguer JF, Hoogenboom M, Ferrier-Pagès C (2016). Heterotrophy promotes the re-establishment of photosynthate translocation in a symbiotic coral after heat stress. Scientific Reports.

[ref-128] Venn AA, Loram JE, Douglas AE (2008). Photosynthetic symbioses in animals. Journal of Experimental Botany.

[ref-129] Wagner DE, Kramer P, Van Woesik R (2010). Species composition, habitat, and water quality influence coral bleaching in southern Florida. Marine Ecology Progress Series.

[ref-130] Warner ME, Fitt WK, Schmidt GW (1999). Damage to photosystem II in symbiotic dinoflagellates: a determinant of coral bleaching. Proceedings of the National Academy of Sciences of the United States of America.

[ref-131] Weston AJ, Chung R, Dunlap WC, Morandini AC, Marques AC, Moura-da-Silva AM, Ward M, Padilla G, Da Silva LF, Andreakis N (2013). Proteomic characterisation of toxins isolated from nematocysts of the South Atlantic jellyfish *Olindias sambaquiensis*. Toxicon.

[ref-132] Weston AJ, Dunlap WC, Beltran VH, Starcevic A, Hranueli D, Ward M, Long PF (2015). Proteomics links the redox state to calcium signaling during bleaching of the scleractinian coral *Acropora microphthalma* on exposure to high solar irradiance and thermal stress. Molecular & Cellular Proteomics.

[ref-133] Williams SM, Sánchez-Godínez C, Newman SP, Cortés J (2016). Ecological assessments of the coral reef communities in the Eastern Caribbean and the effects of herbivory in influencing coral juvenile density and algal cover. Marine Ecology.

[ref-134] Wooldridge SA (2014). Formalising a mechanistic linkage between heterotrophic feeding and thermal bleaching resistance. Coral Reefs.

[ref-135] Yellowlees D, Rees TAV, Leggat W (2008). Metabolic interactions between algal symbionts and invertebrate hosts. Plant, Cell & Environment.

